# Periodontists’ Attitudes and Professional Behavior Towards Surgically Facilitated Orthodontic Tooth Movement—A U.S. National Survey

**DOI:** 10.3390/dj13100468

**Published:** 2025-10-15

**Authors:** John J. Schuetz, Trevor D. Richmond, Mark Scarbecz, Ayman Al Dayeh, Sidney Stein, Vrushali Abhyankar

**Affiliations:** 1 Department of Periodontology, College of Dentistry, University of Tennessee Health Science Center, Memphis, TN 38163, USA; drjohnschuetz@gmail.com (J.J.S.); sstein@uthsc.edu (S.S.); 2 Department of Bioscience Research, College of Dentistry, University of Tennessee Health Science Center, Memphis, TN 38163, USA; mscarbec@uthsc.edu; 3 Department of Orthodontics, College of Dentistry, University of Tennessee Health Science Center, Memphis, TN 38163, USA; aaldayeh@uthsc.edu

**Keywords:** periodontally accelerated osteogenic orthodontics (PAOO), surgically facilitated orthodontic tooth movement (SFOT)

## Abstract

**Background:** Periodontally accelerated osteogenic orthodontics (PAOO) is a surgical procedure to accelerate orthodontic tooth movement and minimize periodontal complications. This study surveyed U.S. periodontists to assess various aspects of the procedure as regards prevalence, training, and execution. **Methods:** The authors developed a unique questionnaire, the first national study of this type, housed on the Qualtrics^®^ survey platform, to analyze trends in PAOO training and use. Unique recruitment emails were sent to 3154 members of the American Academy of Periodontology. 449 U.S. periodontists/3154 surveyed (14.2%) responded to this web-based, anonymized survey. IBM statistical software (SPSS V28) was used for data analysis. **Results:** Among respondents, PAOO training was received during residency (32.7%) and by continuing education (CE) (50.8%), with higher CE (57.3%) by those who did not receive PAOO residency training (*p* < 0.001). 38.5% of periodontists perform PAOO, and those most likely to perform PAOO had both PAOO residency training and CE, with 78.5% performing 1–5 cases/year. Most (87.7%) received 1–2 PAOO referrals/year from orthodontists or general dentists. Differences in techniques and materials were the type of bone graft or membrane used, the position of corticotomies, and the timing of orthodontic movement. The primary PAOO goal was “rapid tooth movement” (41.1%) and to “increase the alveolar housing” (37.2%). The secondary (38%) and tertiary (37.2%) ranked goals were “augment dehiscence or fenestration”, with the “prevention of apical root resorption” ranked as their quaternary goal. **Conclusions:** The results of this survey provide data on the trends, training, and use of PAOO among U.S. periodontists. This information may aid in developing residency curriculum and performing PAOO research.

## 1. Introduction

The number of adult patients who request orthodontic treatment has been increasing with societal awareness of dental function and esthetics. Orthodontics in adult patients is challenging because of slower bone turnover. This leads to increased treatment time and a higher probability of periodontal complications, including fenestrations and dehiscence, recession, and bone loss [1,2]. Globally, research output and clinical interest in surgically accelerated orthodontic techniques such as PAOO, SFOT, and Wilckodontics have grown substantially over the past decade, reflecting the need for efficient and periodontally safe approaches to manage the rising adult orthodontic population [3]. 

Periodontally accelerated osteogenic orthodontics (PAOO) is a surgical technique designed to accelerate orthodontic tooth movement while minimizing periodontal complications. The technique involves applying a series of vertical cuts in the cortical part of the alveolar bone and the application of bone grafts to the area, followed by the application of orthodontic forces. PAOO results in more rapid movement of teeth, improved stability, enhanced buccal bone dimensions, and increased alveolar bone thickness [4,5,6,7,8]. A recent systematic review on surgically accelerated orthodontic techniques reported no significant increase in adverse effects on the periodontium, root resorption, or tooth vitality compared to conventional orthodontics, although transient postoperative pain and swelling were consistently observed [9].

Surgically assisting orthodontic tooth movement with corticotomies started in the 1800s. Kole’s technique [10] involved interdental cuts buccally and lingually through cortical bone and, after raising a full-thickness flap, horizontal cuts over the teeth apices. However, due to the aggressive nature of the technique, it failed to gain widespread acceptance [8]. To retain pulpal and periodontal health, Duker constrained the corticotomy cuts 2 mm short of the alveolar crest [9]. PAOO, the combination of corticotomy cuts with bone grafting, was developed by the Wilcko brothers [11]. The circumscribed injury resulting from the corticotomy incisions led to inflammatory cell recruitment to the injury site and rapidly accelerated the wound’s healing, a process known as Rapid Acceleratory Phenomenon (RAP) [12]. RAP enables orthodontic tooth movement because the bone density at the corticotomy site is decreased and the bone turnover rate is increased [12]. RAP predictably reaches its peak at 1–2 months and lasts about four months. Once the orthodontic treatment is completed, alveolar remineralization ensures enhanced tooth stability [9]. The addition of bone grafting to the corticotomy surgeries helped to retain periodontal health, decrease treatment time, and reduce the risk of root resorption.

Although the PAOO procedure has documented benefits, it does require close collaboration between the orthodontist and the periodontist. The surgical procedure is very invasive and requires a significant training curve to achieve a predictably successful outcome. Not all orthodontic or periodontic residency programs in the United States provide sufficient training to ensure PAOO competence upon graduation. Moreover, there is no single standard treatment regimen due to multiple variations in this procedure. There is negligible data on the use of this procedure among practicing periodontists.

Surveys have been used with success to question dental professionals [13,14,15]. The goals of this study were to use a national survey of U.S. periodontists to: (1) determine the prevalence of PAOO in clinical practice based on residency graduation date and geographic region of training; (2) assess the extent of PAOO training received during residency and/or through continuing education; (3) evaluate variations in surgical techniques and grafting materials utilized; and (4) identify the primary purposes and perceived outcomes of performing PAOO. To our knowledge, no prior research has evaluated these specific parameters in this manner, making this study the first of its kind and offering novel clinical insights to guide evidence-based decision-making for clinicians. The results of this national survey will provide an understanding of the national use and treatment acceptance of PAOO-like surgeries by U.S. periodontists, help standardize the protocol, and determine whether additional training in residency is needed to improve the adoption of this procedure. This information may aid in the research, development, and education of the PAOO surgical protocol. Therefore, this study aimed to survey U.S. periodontists to evaluate the prevalence, training, and execution of PAOO. The null hypothesis was that there are no significant associations between training background and PAOO performance, nor significant differences in techniques, materials, or timing of orthodontic tooth movement among respondents.

## 2. Materials and Methods

### 2.1. IRB Approval

Approval for study 22-08 Survey on periodontally accelerated osteogenic orthodontics (PAOO)945-XM was obtained in 8 September 2022 from the University’s Institutional Review Board (IRB). Participation in the study was voluntary and anonymous. Participation in whole, or in part, indicated consent to participate in the study, and the decision not to participate or stop participating did not result in a penalty or affect your rights as a research subject.

### 2.2. The Survey

The multiple choice cross-sectional survey (which can be found in the online Journal of Periodontology supporting information as “Supplement S1: Questionnaire”) was developed by authors of this study to address the study objectives and variables of interest: (1) the prevalence of PAOO use among practitioners in various US geographic locations; (2) the extent of PAOO training provided during residency and/or by continuing education; (3) variations in surgical technique and materials used; and (4) the purpose and outcomes of performing PAOO. The questionnaire was pretested on periodontal residents and authors. The questionnaire was located at the Qualtrics^®^ survey platform, and its software applied question-specific built-in validation (custom validation, content, or question-specific validation) as appropriate for each question and reported validated results.

### 2.3. Survey Population, Survey Distribution

Unique recruitment emails were sent to 3154 active (in clinical practice) members of the American Academy of Periodontology. This includes 2158 from Datacaptive.com and 996 from the Centers for Medicare and Medicaid Service’s National Plan and Provider Enumeration System (NPPES). We received 449 responses with 443 valid responses.

Following best practices regarding web-based surveys, periodontists received an invitation letter explaining the study with the consent form and survey link [16,17,18,19,20,21]. The survey was available on mobile or computer via Qualtrics. Emails were individualized to track completion and target non-responders with reminders at 1, 2, 3, and 4 weeks after the first invitation.

### 2.4. Confidentiality and Security

Respondent’s anonymity and confidentiality were ensured using Qualtrics^®^ web-based secure survey platform under a license with the University. Only the principal investigators had authorized access to the data. Each respondent received an anonymous individualized URL link, ensuring that no personally identifiable information was collected by the system. Because the link was individualized, the program was able to send selective reminders to non-participants. The data were downloaded, stripped of unnecessary variables, saved as an SPSS V28 (statistical software, IBM, Armonk, NY, USA) data file, and securely stored on a password-encrypted computer available only to the study investigators.

### 2.5. Survey Data Analysis

Survey data were analyzed using IBM V28 SPSS (Armonk, NY, USA) statistical software licensed by the University to determine statistical significance, including cross-tabulation analysis and Chi-Square Tests of Independence. Statistical significance was set at *p* < 0.05. Missing data were minimal and excluded from the analysis.

## 3. Results

### 3.1. Demographic Data

Four hundred forty-nine (449) periodontists responded to the survey, with 413 indicating the year they finished their residency training, ranging from 1970 to 2022 (Table 1). Responses were highest among the more recent graduates. Four hundred thirty periodontists indicated the AAP district/state where they completed their periodontal residency, and the residency locations were broadly geographic (Table 1).

### 3.2. PAOO Training During Residency and by Continuing Education

Only 32.7% of respondents received PAOO training during their residency (Table 2). Cross-tabulation analysis found no significant difference in PAOO residency training based on the AAP district of training (*p* = 0.2434) (Supplement Table S1). Based on the current geographic distribution of AAP periodontists in the US, our survey sample closely aligned with where periodontists are practicing nationwide. But data analysis showed PAOO residency training increased with each successive decade (Table 3). For example, only 24% of respondents were taught PAOO during 2000–2009 residencies versus 68.4% of respondents between 2010 and 2019.

Post-residency, 50.8% of all respondents pursued continuing education (CE) (Table 2). However, of those who had PAOO training during their residency, only 37.4% still followed up with further PAOO CE (Table 3), while among those without PAOO residency training, more than half (57.3%) pursued PAOO CE. This difference suggests periodontists without PAOO training realized they needed PAOO CE while in practice.

### 3.3. Percentage of Periodontists Performing PAOO

The survey revealed that 38.5% (170) of respondents perform PAOO in their practice, while 61.5% (272) do not (Table 2). This may reflect, in part, that only 14% received referral requests for PAOO (Table 2). Cross tabulation analysis indicated there was no relationship between the AAP district of graduation and whether they performed PAOO (*p* = 0.890). However, cross-tabulation analysis found periodontists trained in district 3 (AL, FL, GA, KY, MS, NC, Puerto Rico, SC, TN, U.S. Virgin Islands, VA) were the most likely to perform PAOO (*p* = 0.025). (Supplement Table S1)

Among the periodontists who received PAOO residency training, those who attended CE were the most likely to perform PAOO (69.1%), while only 37% of those without additional CE performed PAOO (Table 4). Those with neither PAOO training nor CE were the least likely to practice PAOO (87.1%). Among those who did not have PAOO in residency, but took PAOO CE, they were about equally likely to carry out PAOO in office (48.2%) or not (51.8%). Surprisingly, 12.9% of periodontists reported performing PAOO without formal PAOO training or CE.

### 3.4. PAOO Practice Statistics

When respondents were asked to give a single reason, they were not performing PAOO. The primary reasons given were lack of referrals (56.3%), insufficient training (18.5%), disbelief in the procedure (6.7%), and lack of procedure awareness (2.2%) (Table 5). Among the 170 respondents performing PAOO, most (78.5%) performed 1–5 PAOO cases per year, 11% performed 6–10 cases, and only 3.1% performed 21 cases or more cases annually (Table 5). The majority (87.7%) received 1–2 referrals per year from orthodontists or general dentists, with only 1.3% of periodontists receiving 5–7 or greater referrals per year (Table 5).

### 3.5. Variation in Surgical Techniques and Materials Used

Among the 170 respondents performing PAOO, only 9.2% reported using a flapless technique (Table 6). The majority (74.1%) of periodontists used corticotomies to harness the regional acceleratory phenomenon (Table 6). The most common corticotomy cuts used were deep cortical and medullary extending interdentally in the coronal-apical direction (33.5%) or deep cortical and medullary interdentally and apically (30.4%), with fewer periodontists using cuts superficial to the cortical bone only extending interdentally in the coronal-apical direction (18.6%) or superficial cortical bone only interdentally and apically (17.4%). They most commonly stop coronal interdental corticotomy cuts 1–2 mm apical to CEJ (36.4%), near but below the CEJ (23.5%), or at the crest or slightly past the crest (21%). Only 15.4% stop interdental corticotomy cuts 3 mm or more apical to CEJ.

Most periodontists used Allograft (50%), a combination of Allograft and Xenograft (39.5%), or Xenograft (8.6%) bone graft materials (Table 6).

Twenty-nine (29.2) percent of respondents performing PAOO reported not using a membrane. Among the respondents who use membrane, the majority (60.9% of total respondents) reported using collagen membrane. The remaining 9.9% reported using AlloDerm or similar membrane (9.3%) or a non-resorbable membrane (0.6%) (Table 6). Cross-tabulation analysis similarly showed that regardless of whether they were using allograft bone graft, xenograft, or a combination of those, they were most frequently paired with a resorbable collagen membrane > no membrane > AlloDerm membrane (Table 6).

The majority of respondents performed augmentation on buccal surfaces (65.4%), with 28.4% varying their approach depending on the referral, and 6.2% augmenting buccal and lingual surfaces (Table 6).

Most periodontists (44.7%) indicated that the stage of orthodontic treatment they prefer to perform PAOO depends on the case, 38.5% after placing brackets but before placing the wire, 11.8% reported at any stage of orthodontic treatment, and 5% indicated they prefer to perform PAOO before placing brackets (Table 6).

### 3.6. Primary Goal or Outcome for PAOO

When respondents performing PAOO were asked to rank the PAOO primary goal or outcome, the primary goals were “rapid tooth movement” (41.1%) and to “increase the alveolar housing” (37.2%). The secondary and quaternary goals focused on “augment dehiscence or fenestration” and “prevention of apical root resorption”, respectively (table not needed- data is self explanatory).

## 4. Discussion

Extensive research indicates that PAOO used to accelerate orthodontic treatment improves both periodontal outcome and decreases the duration of patient treatment [7]. However, to our knowledge, no prior survey assessed U.S. periodontists’ PAOO training, knowledge, and practice patterns. Web-based surveys are cost-effective but may have a lower response rate than some other methods [1,16], which was true of this study.

PAOO training in residency has increased steadily with each successive decade, with an exponential rise observed after the year 2000. While PAOO was formally introduced in 2001 by Drs. Thomas and William Wilcko [12], some respondents reported receiving training before this. This is notable, given that the term PAOO and the patented “Wilckodontics” procedure had not yet been introduced. It is plausible that these respondents indicated ‘yes’ to receiving PAOO training because they were trained in surgically facilitated orthodontic therapy (SFOT). This could also explain the heterogeneity observed in our results regarding variations in surgical technique, such as flapless versus flap approaches, use of membranes, corticotomy cut locations and types, timing within orthodontic treatment, and bone graft materials used.

Awareness of PAOO among periodontists has grown substantially, as evidenced by 74.4% of respondents who graduated in 2020 or later indicating that their residency programs included PAOO training. According to the 2025 AAO Member Census, approximately 28.7% of orthodontic patients in the United States are adults, representing nearly 1.91 million individuals in active treatment, and the availability of continuing education in PAOO, the trend towards incorporating PAOO training into periodontal residency programs is progressing positively [22].

The rise in PAOO procedures correlates with the increased number of adults seeking orthodontic treatment today, attributable to several patient-related factors. The widespread use of social media and heightened societal emphasis on dental aesthetics have significantly raised awareness about the importance of a well-aligned smile. Additionally, the increased accessibility of orthodontic treatments, in terms of both availability and affordability, has further contributed to this trend. Many adults who did not receive orthodontic care during adolescence are now seeking treatment, driven by the longer retention of natural dentition and a desire to improve oral health and function [1]. Furthermore, with the advancements in discreet orthodontic technologies (e.g., clear aligners), coupled with increasing awareness of oral health and esthetics among adults, have significantly contributed to the rising demand for adult orthodontic treatment in recent years [23]. The growing number of residency programs teaching PAOO and the availability of continuing education courses have also contributed to heightened clinician awareness and advocacy for the procedure and enabling more practitioners to offer this advanced treatment.

How generalizable are these results? The survey demonstrated a broad cross-section across AAP districts. While the exact representativeness of the sample cannot be determined, the geographic distribution suggests a reasonable degree of external validity. While our survey found no significant association between geographic location and PAOO practice frequency, practitioners in AAP districts three and four were more likely to perform PAOO, suggesting regional differences in training emphasis and clinical practice patterns.

Respondents with both PAOO residency training and PAOO CE were the most likely to perform PAOO in their practice, while those without either form of training were the least likely. There is a strong association between attending PAOO continuing education courses and performing the procedure. However, causality is difficult to infer—whether practitioners attend CE courses because they already perform the procedure, or they perform the procedure because they attended the course. The likelihood of attending PAOO CE after residency depended on whether the practitioner had received PAOO training during residency. This suggests that periodontists without PAOO residency training recognized the need for PAOO CE while in practice. Concerningly, 12.9% of periodontists reported performing PAOO without any formal training in residency or through a CE course, which underscores the need for standardized training and certification.

There is considerable heterogeneity in PAOO protocols, including variation in flap design, depth, and number of corticotomy cuts, graft use, and timing of orthodontic activation [19]. Our results revealed significant variation in surgical techniques, including flapless versus flap approaches, use of membranes, corticotomy cut locations and types, timing within orthodontic treatment, and bone graft materials used. This high degree of variation may be attributable, in part, to the diverse nomenclature for the procedure, such as surgically facilitated orthodontic therapy, accelerated osteogenic orthodontics (AOO), periodontally accelerated osteogenic orthodontics (PAOO), corticotomy-assisted orthodontic treatment (CAOT), selective alveolar decortication (SAD), and corticotomy-facilitated orthodontics (CFO). SFOT was introduced into the field more than a century before PAOO, leading to significant modifications in surgical procedures over time. Practitioners from the 1970s through the 1990s who indicated they were taught PAOO likely encountered different delineations in corticotomies, use of flaps, membranes, and bone graft materials. This is further supported by recent literature describing multiple modified corticotomy techniques, including corticision, piezocision, discision, and micro osteoperforation, which vary in invasiveness and whether a flap is required [24,25]. The lack of standardization in PAOO procedures inherently leads to variability in how practitioners perform the surgical protocol, contributing to the observed diversity in our results and emphasizing the need for uniform protocols. This suggests that programs need to expose the residents to this procedure during their training to ensure a better adoption of PAOO.

The fact that 65.4% of respondents indicated that they perform PAOO only on buccal surfaces may suggest a higher prevalence of orthodontic cases requiring expansion rather than arch closure. Although no respondent selected lingual/palatal augmentation surfaces, this does not imply that these procedures are never performed. This inference can be primarily supported by the answer option “depends on referral”. Moreover, although bone thickness typically increases after PAOO, the magnitude and duration of this change depend on the selected bone graft material, whether augmentation is performed labially or both buccally and lingually, and whether soft tissue augmentation or phenotype modification is included [20].

The survey results provide insight into the clinical decision-making process of practitioners, highlighting the multifaceted goals they balance when performing PAOO. The practitioner’s primary goals for PAOO—rapid tooth movement and increasing alveolar housing—reflect both patient and practitioner priorities. Both primary goals highlight the dual emphasis on improving structural support for teeth and expediting orthodontic treatment timelines, reflecting the core benefits of PAOO. It suggests that residency programs and CE courses should emphasize techniques for increasing alveolar housing and rapid tooth movement, while also covering the management of dehiscence, fenestration, and root resorption. The variability in goals and the high ranking of “other” for quinary goals reflect the diverse applications and potential benefits of PAOO. This indicates a need for further research to establish standardized protocols and guidelines that can encompass the full spectrum of practitioner objectives and patient needs.

Despite providing valuable insights into the trends and variations in PAOO practice and training, our study has several limitations. First, the survey data relies on self-reported information, which may introduce bias or inaccuracies in reporting training and practice patterns. Second, the lack of a standardized definition and nomenclature for PAOO procedures across different regions and training programs may have contributed to the observed heterogeneity in surgical techniques and materials. Third, our analysis did not account for the potential influence of differing referral patterns on the likelihood of performing PAOO, which complicates the interpretation of geographic variations. Fourth, while our study highlights the importance of both residency and continuing education, it does not assess the quality or content of these training programs, which could vary significantly. Lastly, the cross-sectional nature of the survey limits our ability to draw causal inferences about the relationship between training and PAOO practice.

A low response rate (RR) relative to the overall U.S. periodontists is also a limitation of the study. Declining participation in epidemiological surveys—particularly those conducted online—is a well-documented trend. Although web-based surveys offer cost-effective advantages, they often yield lower response rates compared to other methods. These rates can vary depending on both the survey topic and the population being studied. Given the nature of this survey, the low RR is not unexpected as PAOO is a relatively newer procedure in everyday periodontal practice.

Future research should aim to address these limitations by incorporating longitudinal data, standardized definitions, and a more comprehensive evaluation of training program content and quality. Since the procedure itself is dependent on referrals from orthodontic colleagues, a similar survey of the orthodontists’ awareness and acceptance of the procedure would be beneficial. We were not able to assess the demographics of the populations (young, middle-aged, old) of PAOO being performed. It would have been beneficial to know the age group that was most frequently being referred for the PAOO procedure.

## 5. Key Conclusions

- PAOO has shown a rising prevalence among recent graduates since post-2000 due to an increase in adult orthodontics
- Training Trends
  ○ There is growth in residency programs incorporating PAOO after 2000.
  ○ Comprehensive training (residency + CE) is strongly linked to the likelihood of performing PAOO.
- Geographic and Referral Dynamics
  ○ Geographic variation in PAOO practice is observed; however, Residency location is not a direct predictor of practice region.
  ○ Referral patterns play a critical role in PAOO adoption.
- Technique and Material Diversity
  ○ There is a wide variation in surgical approaches and materials used, reflecting revolving nomenclature and historical development, indicating the need for greater procedural standardization.
- Clinical Goals and Patient Expectations
  ○ Primary goal of PAOO is to increase alveolar housing and accelerate tooth movement, which aligns with patient demand for faster, effective orthodontic care.
  ○ Training programs should address the full spectrum of procedural aspects.
- Interdisciplinary Education Emphasis
  ○ CODA highlights the importance of interdisciplinary care.
  ○ Residency programs should integrate collaborative training models.
- Call for Standardization and Research
  ○ Findings underscore the need for standardized protocols.
  ○ Further research is essential to optimize PAOO outcomes.

## Figures and Tables

**Table 1:** Respondents’ Demographics: Decade and AAP district of periodontal residency.

Categorization by Years Completed Residency	Respondents (N)	CUMULATIVE % by Category
1970s	44	10.7%
1980s	60	14.5%
1990s	74	17.9%
2000s	75	18.1%
2010s	117	28.5%
2020–2022	43	10.4%
**AAP district/state**	**Valid %**	N
District 1: CT, ME, MA, NH, RI, VT	9.3	40
District 2: DE, DC, MD, PA, WV, VA	11.6	50
District 3: AL, FL, GA, KY, MS, NC, Puerto Rico, SC, TN, U.S. Virgin Islands, VA	16.5	71
District 4: IL, IN, IA, KS, MI, MN, MO, ND, OH, SD, WI	18.8	81
District 5: AR, CO, LA, NE, OK, TX	13.5	58
District 6: AK, AZ, CA, HI, ID, MT, NV, NM, OR, UT, WA, WY	13.5	58
District 7: NJ, NY	8.6	37
District 8: Federal Dental Services	8.1	35
Total Respondents	100	430

**Table 2:** Respondents PAOO training, performance, and referrals.

	% YES	N	% NO	N	% Unknown	N	Total N
Percentage of respondents taught PAOO during their residency	32.7	147	66.1	293	0.7	3	443
Percentage of respondents who attended PAOO continuing education since graduation from residency	50.8	225	49.2	218			443
Percentage of respondents who perform PAOO in their office	38.5	170	61.5	272			442
Percentage of respondents with referral requests for PAOO	14.0	38	86.0	233			271

**Table 3:** Respondents taught PAOO by graduation year.

		Taught PAOO in Your Residency?						
**Grad yr**	**Yes**			**No**		**N**		
1970–1979	2.3%			97.7%		43		*p* < 0.001
1980–1989	1.7%			98.3%		59		
1990–1999	5.4%			94.6%		74		
2000–2009	24.0%			76.0%		75		
2010–2019	68.4%			31.6%		117		
2020–2022	74.4%			25.6%		43		
**PAOO training in residency and by CE**								
		**PAOO CE**						
**PAOO in Residency**		**Yes**			**No**		**N**	
**Yes**		37.4%			62.6%		147	
**No**		57.3%			42.7%		293	
**N**		223			217			
**PAOO training by residency and CE by graduation year**								
			**PAOO CE**					
**PAOO in Residency**	**Grad yr**		**Yes**		**No**	**N**		
**Yes**	1970–1979		100.0%		0.0%	1		*p* = 0.024
	1980–1989		0.0%		100.0%	1		
	1990–1999		50.0%		50.0%	4		
	2000–2009		44.4%		55.6%	18		
	2010–2019		48.8%		51.2%	80		
	2020–2022		15.6%		84.4%	32		
			40.4%		59.6%	136		
**No**	1970–1979		47.6%		52.4%	42		*p* = 0.003
	1980–1989		58.6%		41.4%	58		
	1990–1999		72.9%		27.1%	70		
	2000–2009		61.4%		38.6%	57		
	2010–2019		45.9%		54.1%	37		
	2020–2022		18.2%		81.8%	11		
			57.8%		42.2%	275		

**Table 4:** Respondents PAOO Training in residency, CE, performing now.

		Perform PAOO Now		
Taught PAOO in Residency	PAOO CE	Yes	No	N
Yes	Yes	69.1%	30.9%	55
	No	37.0%	63.0%	92
	N	72	75	
No	Yes	48.2%	51.8%	168
	No	12.9%	87.1%	124
	N	97	195	

*p* < 0.001.

**Table 5:** PAOO practice statistics.

Reasons Periodontists Do Not Perform PAOO	Valid %	N
No referring providers	56.3	152
Lack of appropriate training	18.5	50
Do not believe in the procedure	6.7	18
Not aware of the procedure	2.2	6
Non-specified reasons	16.3	44
Total responses		270
**PAOO cases performed per year**	**Valid %**	**N**
None	5.5	9
One–Five	78.5	128
Six–Ten	11	18
Eleven–Fifteen	0.6	1
Sixteen–Twenty	1.2	2
Twenty-One or More	3.1	5
Total responses		163
**# Referrals by orthodontists or general dentists for PAOO**	**Valid %**	**N**
One to Two	87.7	136
Three to Four	9.7	15
Five to Six	1.3	2
Seven or Greater	1.3	2
Total responses		155

**Table 6:** Variations in surgical technique.

Technique Used to Accelerate Tooth Movement	Valid %									
	YES	N			NO			N		
Flapless technique	9.2	15			90.8			148		
Corticotomy/bone grafting technique	74.1	120			25.9			42		
**Type of corticotomy cuts used**	**Valid %**	**N**								
Deep cortical and medullary extending interdentally in the coronal-apical direction	33.5	54								
Deep cortical and medullary interdentally and apically	30.4	49								
Cuts superficial cortical bone only extending interdentally in the coronal-apical direction	18.6	30								
Superficial cortical bone only interdentally and apically	17.4	28								
**Corticotomy cut stopping point**	**Valid %**	**N**								
1–2 mm apical to CEJ	36.4	59								
Near but below CEJ	23.5	38								
At the crest or slightly past the crest	21	34								
3 mm or more apical to CEJ	15.4	25								
**Bone graft substitute**	**Valid %**	**N**								
Allograft	50	81								
Xenograft	8.6	14								
Combination	39.5	64								
None of the above	1.9	3								
**Membrane type**	**Valid %**	**N**								
Resorbable Collagen	60.9	98								
Alloderm or Similar Membrane	9.3	15								
No Membrane	29.2	47								
Non-Resorbable Membrane	0.6	1								
	**Bone material used in majority of cases**									
**What membrane do you use in majority of cases**	**Allograft (%)**	**N**	**Xeno-graft (%)**	**N**	**Combo (%)**	**N**	**Other (%)**	**N**	**Total %**	**Total**
No membrane	32.5	26	7.1	1	28.1	18	66.7	2	29.2	47
Non-resorbable	1.3	1	0	0	0	0	0	0	0.6	1
Resorbable collagen	56.3	45	85.7	12	62.5	40	33.3	1	60.9	98
Alloderm or similar	19	8	7.1	1	9.4	6	0	0	9.3	15
Total	100	80	100	14	100	64	100	3	100	161
**Surface where augmentation is performed**	**Valid %**	**N**								
Only buccal	65.4	106								
Always buccal and lingual/palatal	6.2	10								
depends on referral	28.4	46								
**Stage of orthodontic treatment when performing PAOO**	**Valid %**	**N**								
Before placing brackets	5	8								
After placing brackets but before placing the wire	38.5	62								
At any stage of orthodontic treatment	11.8	19								
It depends on the case	44.7	72								

## Data Availability

The original contributions presented in this study are included in the article and Supplementary Material. Further inquiries can be directed to the corresponding author.

## Supplementary Materials

The following supporting information can be downloaded at: https://www.mdpi.com/article/10.3390/dj13100468/s1, Table S1: AAP district of graduation and PAOO; Supplement S1: Questionnaire for Periodontally accelerated osteogenic orthodontics (PAOO).
