# Supplementary material for: Periodontists’ Attitudes and Professional Behavior Towards Surgically Facilitated Orthodontic Tooth Movement—A U.S. National Survey
Source: Dent J (Basel). 2025 Oct 15;13(10):468. doi: 10.3390/dj13100468 (PMC12564588; doi:10.3390/dj13100468)
Supplement: Supplementary file 1 [file dentistry-13-00468-s001.zip › dentistry-3872057-supplementary-Questionnaire.pdf]

Supplement 1: Questionnaire for Periodontally accelerated osteogenic orthodontics (PAOO)

1. What year did you finish your residency training?
   Drop down box with years from 1970-2022

2. What AAP district/state did you **graduate periodontal residency** from?
   a. District 1: ME, VT, NH, MA, CT, RI
   b. District 2: NY
   c. District 3: NJ, PA, MD, DE, DC, MD
   d. District 4: KY, TN, VA, NC, SC
   e. District 5: MI, OH, IN
   f. District 6: ND, SD, NE, KS, MN, IA, MO, WI, IL
   g. District 7: TX, OK, AR, LA, MS
   h. District 8: WA, OR, ID, MT, WY, NV, UT, CO, AZ, NM
   i. District 9: CA
   j: District 10: AL, GA, FL

3. Were you taught PAOO in your periodontal residency program?
   a. Yes
   b. No
   c. Unknown

4. Have you attended continuing education about PAOO since graduation from residency?
   A. Yes
   B. No

5. Do you perform PAOO in your office?
   A. Yes
   B. No

Questions for those who don't perform PAOO
6. Has a referral ever requested you to perform this procedure?
   A. Yes
   B. No

7. What reason are you not performing this procedure?
   a. not aware of this procedure
   b. Lack of appropriate training
   c. No referring providers
   d. Do not believe in the procedure
   e. Other:

Questions for those who do perform PAOO
6. How many cases do you perform a year?

a. None
b. 1-5
c. 6-10
d. 11-15
e. 16-20
f. 21 or more

7. In the last year, how many orthodontists or general dentists refer to you for PAOO?
a. 1-2
b. 3-4
c. 5-6
d. 7 or greater

8. What bone materials do you use with the majority of your cases?
a. Allograft
b. Xenograft
c. Alloplast
d. Combination
e. None of above

9. Have you performed the flapless technique?
a. Yes
b. No

10. Do you always place bone with corticotomies?
a. Yes
b. No

11. What membrane do you use with the majority of your cases?
a. No membrane
b. non-resorbable
c. Resorbable collagen
d. Alloderm or similar
e. Xenoderm or similar
If you wish to justify your answer:

12. What statement describes your corticotomy cuts best?
a. Superficial cortical bone only extending interdentally in the coronal-apical direction
b. Superficial cortical bone only interdentally and apically
c. Deep cortical and medullary extending interdentally in the coronal-apical direction
d. Deep cortical and medullary interdentally and apically

13. Where do you stop coronoal interdental corticotomy cuts
a. At the crest or slightly past the crest

b. Near but below CEJ
c. 1-2mm apical to CEJ
d. 3mm or more apical to CEJ
e. doesn’t apply

14. What surfaces do you perform augmentation
    a. Only Buccal
    b. Only Lingual/palatal
    c. Always buccal and lingual/palatal
    d. Depends on referral

15. At what stage of orthodontic treatment do you prefer to perform the PAOO
    a. Before placing the brackets
    b. After placing brackets but before placing the wire
    c. At any stage of orthodontic treatment
    d. It depends on the case
    e. Does not apply

16. What is your primary goal or outcome (maybe a rank order question)
    a. augment dehiscence or fenestration
    b. increase the alveolar housing
    c. rapid tooth movement
    d. prevention of apical root resorption
    e. other:
